# Safety incidents during intrahospital transport of hemodialysis patients in China: a multicenter cross-sectional study

**DOI:** 10.3389/fpubh.2026.1932458

**Published:** 2026-08-18

**Authors:** Jing Li, Yao Liu, Liyun Cao, Meng Zhao, Huipeng Xing, Yinbilige Se, Yuqing Chen

**Affiliations:** 1 Renal Division, Department of Medicine, Peking University First Hospital, Beijing, China; 2 Institute of Nephrology, Peking University, Beijing, China; 3 Key Laboratory of Renal Disease, Ministry of Health of China, Beijing, China; 4 Key Laboratory of Chronic Kidney Disease Prevention and Treatment, Ministry of Education, Beijing, China

**Keywords:** hemodialysis, intrahospital transport, medical quality, patient safety incident, renal replacement therapy, transition of care

## Abstract

**Objective:**

To characterize safety incidents during intrahospital transport of hemodialysis patients in Chinese tertiary hospitals to guide evidence-based safety standards.

**Methods:**

A multicenter cross-sectional study (STROBE-guided) included 4,253 registered nurses consisting of 418 head nurses and 3,835 clinical nurses. While the WHO’s International Classification for Patient Safety served as the basis for the classification of safety incidents, the structured questionnaires were created based on clinical observation and a review of the literature. Binary logistic regression identified organizational and operational risk factors.

**Results:**

Over 12 months, 268 safety incidents were reported from 135,459 intrahospital transports. Hemodialysis facilities accounted for 81.72% reports. Survey report from 3,232 clinical nurses showed that the predominant incident categories were medical device/equipment malfunctions (61.14%), patient accidents (45.24%), blood/blood product-related events (43.94%), oxygen/gas/vapor issues (42.39%), and clinical administration errors (41.83%). Key independent risk factor was a monthly hemodialysis caseload exceeding 3,000 procedures (OR = 2.580; 95% CI: 1.198–5.556; *p* = 0.015). Additionally, enhanced incident detection rates, facilitated by proactive reporting initiatives, showed a robust positive association with the efficacy of quality control measures (OR = 3.540; 95% CI: 1.604–7.814; *p* = 0.002).

**Conclusion:**

High-volume dialysis units are at significantly elevated risk for transport safety incidents. Units implementing quality control measures reported more safety incidents, which likely reflects the role of a proactive reporting culture in enhancing the detection of hidden incidents. These findings support targeted resource allocation and policy standardization for safer intrahospital transport in hemodialysis settings.

## 1 Introduction

Transitions of care encompass the various points where a patient moves to, or returns from, a particular physical location or makes contact with a health care service for the purposes of receiving health care (1). Optimal patient safety relies heavily on the seamless coordination of care during transitions, and intrahospital transport (IHT) represents a critical yet frequently understudied component, except when involving patients in critical condition, children, or surgical patients (2). The Institute for Safe Medication Practices guidelines for the safe transfer of patients requiring IHT explicitly classify dialysis patients, patients receiving vasoactive medications, and intubated patients as high-acuity transfers (3). The International Classification for Patient Safety (ICPS), published by the World Health Organization (WHO) (4) identifies these transition points as high-risk intervals where the continuity of monitoring and intervention is most vulnerable. Under this framework, a patient safety incident (PSI) is defined as any event or circumstance that could have resulted, or did result, in unnecessary harm to a patient and crucially encompassed adverse events (harmful incidents resulting in injury), no-harm incidents (errors detected prior to reaching the patient or causing damage), and near misses (events intercepted before potential exposure to harm) (5). By standardizing the taxonomy of patient safety issues, the ICPS framework facilitates rigorous comparative analysis across institutions and timeframes, highlighting the imperative to mitigate risks during dynamic care processes.

Maintenance hemodialysis (MHD) patients present physiological vulnerabilities during IHT that exhibit a more complex condition profile than general patients yet require less intensive life support and monitoring than critical ill patients. Specifically, hospitalization for unexpected deterioration was more common among hemodialysis patients than in the ordinary population (15.6–36.8% vs. 0.9–3.4%) (6), and the actual transit process was fraught with additional challenges, such as a heavy reliance on life-sustaining medical equipment, strict infection control requirements, and complicated information handovers (7). Adopting general patient transport regulations may fail to address dialysis-specific requirements such as equipment priming, anticoagulant scheduling, fluid volume management, vascular access care, and dialysis parameter continuity (7, 8). Furthermore, MHD patients need repeated, cyclical transfers between their original wards and the hemodialysis center throughout their hospital stay, in contrast to critically ill patients who typically undergo single-episode transports for acute procedures. This pattern accumulates risk over time (6, 7) yet currently receives insufficient clinical attention. When applied to MHD patients, standardized ICU-based protocols may result in severe medical resource waste (2) and safety gaps due to misalignment with chronic dialysis pathophysiology (8, 9). Patient safety during IHT procedures may be further jeopardized by other unanticipated events, such as extended transit delays, equipment malfunctions, personnel shortages, poor coordination, non-standardized handover protocols, and insufficient transport preparation (10, 11). Regardless of specific scenarios, any risk factor during transport may cause hemodynamic instability characterized by fluid shifts and electrolyte disturbances inherent to renal replacement therapy during times of reduced monitoring outside specialized hemodialysis units, including profound hypotension, malignant arrhythmias, or cardiac arrest (12, 13).

Despite these increased hazards, the majority of existing transition regimens were designed for critically ill or pediatric patients (2, 14). Standard handover checklists, which have been demonstrated to be effective in lowering adverse events in ICU settings (15), frequently ignore crucial hemodialysis-specific procedures such as dialysis machine priming checks or anticoagulant timing relative to transport windows. Moreover, the healthcare delivery landscape varies significantly across regions. In developed nations, over 80% of MHD services are provided through independent dialysis facilities that necessitate complicated inter-hospital transports (9), whereas developing nations (including some regions of Asia) predominantly utilize in-hospital dialysis centers requiring regular intra-hospital shuttles (6). Therefore, it is important to proactively establish safety strategies for various transit phases in a range of clinical settings. The safety burden is substantial yet inadequately quantified in China, where hospital-based hemodialysis remains the most prevalent service paradigm, adherence to general IHT standards is heterogeneous, and awareness of systematic risk prevention is limited (6, 16).

To safeguard patient safety, a standalone transit organization designed especially for MHD patients is desperately needed (8). This organization could integrate multidisciplinary collaboration between dialysis clinicians and general hospital staff, utilize validated assessment checklists, and adhere to evidence-based quality improvement frameworks. Medical resources can be distributed more scientifically and logically by evaluating travel hazards and putting tiered transport protocols into place. This will optimize “green transport” outcomes for that vulnerable population (17). As everyone is aware, nurse engagement in patient safety initiatives has demonstrated measurable benefits in preventing or minimizing PSIs and mitigating possible dangers in healthcare environments. Systematic collection and utilization of nurse-reported safety incident data represent a critical pathway for improving transition-of-care services.

This study aimed to thoroughly describe the nature and type of IHT-related safety incidents involving hemodialysis patients from the viewpoint of nurses. Beyond descriptive epidemiology, we intend to identify practical safety indicators that can guide the formulation of a dedicated risk-stratification tool within the ICPS conceptual framework. In complex hospital environments, it can improve patient outcomes and optimize the delivery of renal replacement therapy by strengthening the resilience of care pathways for MHD patients.

## 2 Methods

### 2.1 Study design

This study employed a multi-center cross-sectional survey to conduct a descriptive analysis of the particular profile of safety incidents associated with IHT among hemodialysis patients at tertiary hospitals in mainland China from June to July 2025. The Strengthening the Reporting of Observational Studies in epidemiology (STROBE) statement (18) was followed in its reporting. Two structured questionnaires were created using a modified Delphi approach for gathering information on the clinical practice of IHT involving hemodialysis patients.

### 2.2 Setting and sample

**Figure 1:**
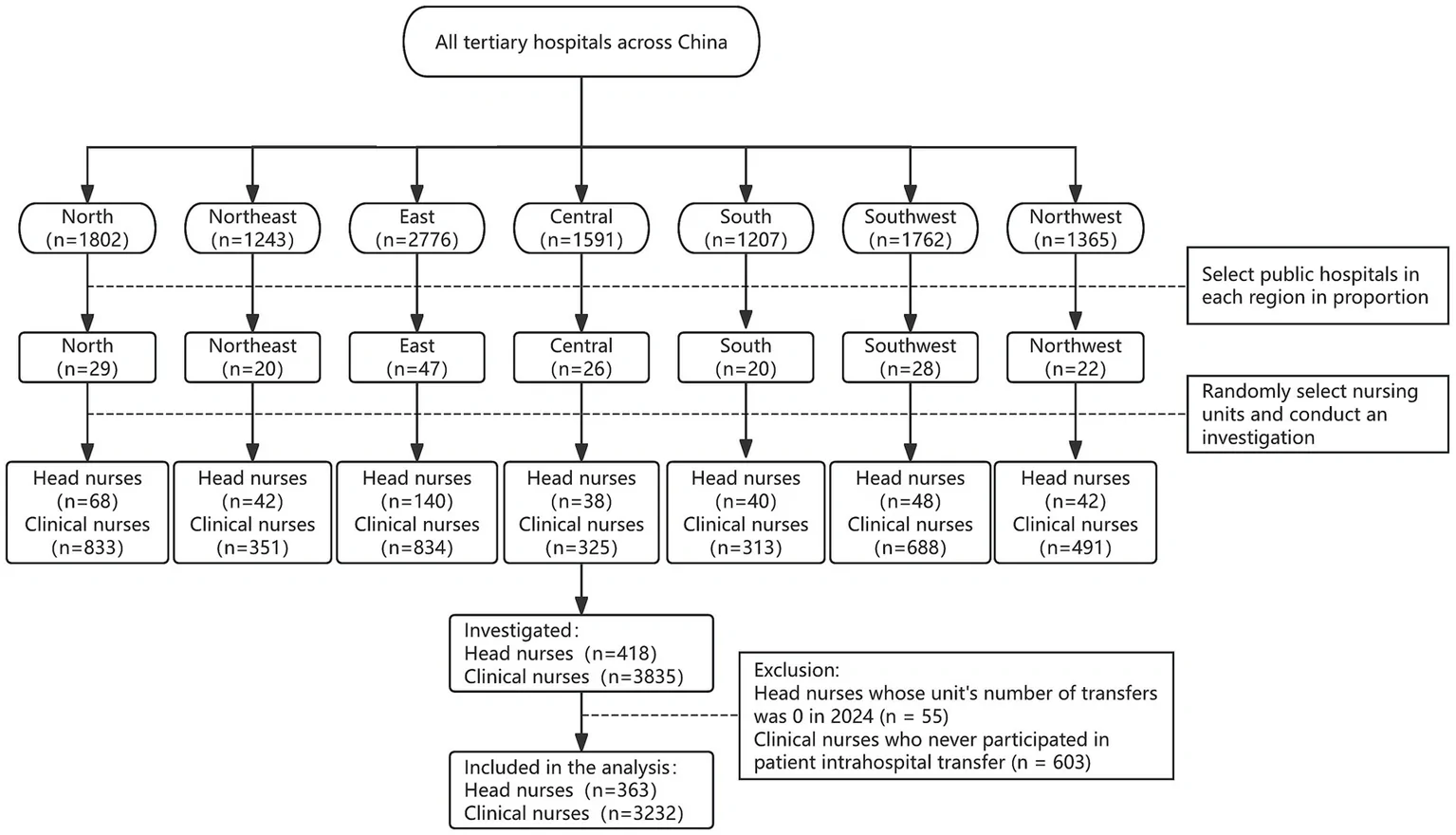
Sample selection process.

A multistage sampling strategy was employed to ensure thorough geographic coverage. At Stage 1, the 11,772 public hospitals in the national database were stratified into seven geographical regions: Eastern, Southern, Central, Northern, Northwestern, Southwestern, and Northeastern. Stage 2 utilized proportional allocation sampling, selecting tertiary hospitals performing intrahospital hemodialysis patient transfers at a regional-to-institution ratio of roughly 1:60. In Stage 3, at least two units from each hospital were randomly chosen, comprising both transmitting units (such as general wards, departments of emergency, and ICU) and receiving units (hemodialysis centers). Head nurses and clinical nurses from particular units finished the investigation in Stage 4. Since each unit had only one head nurse, the total number of head nurses was identical to the number of units surveyed. The sampling process is depicted in Figure 1.

There were 10,221 clinical nurses working in all of the participating units. The sample size was calculated using Cochran’s formula for finite populations (19): n = [Z^2^ × p(1-p)]/e^2^, with *Z* = 1.96 (95% confidence level), *p* = 0.5, and e = 0.05 (margin of error). This yielded the requisite minimum sample size of 385 individuals. The final sample size was calculated as follows: effective sample size/(1 - non-response rate anticipated) = 514 respondents, with an extra 25% buffer added to allow for expected non-response (20). Ultimately, 4,253 questionnaires were distributed (418 to head nurses and 3,835 to clinical nurses). After eliminating incomplete or inconsistent replies, 3,232 (84.28%) valid clinical nurse surveys and 363 (86.84%) valid head nurse surveys remained for analysis.

Head nurse inclusion criteria were (1) ≥ 1 year in nursing management roles and (2) informed consent provided. Exclusions comprised (1) non-frontline administrative positions (e.g., nursing department directors) and (2) absence >3 months during the 6-month data collection window. Clinical nurse inclusion criteria were (1) ≥ 1 year of clinical practice experience and (2) involvement in hemodialysis patient transfers. Exclusions applied to (1) non-assigned personnel (visiting nurses or trainees) and (2) absent >3 months in the prior 6 months for training or other reasons.

### 2.3 Instrument

#### 2.3.1 Development of questionnaires

Initial drafts were derived from systematic literature review and clinical observation of hemodialysis patient transfer practices. Seven experts from academics, clinical management, and clinical nursing participated in a two-round modified Delphi process to establish content validity (21). Five experts (71.43%) had master’s degrees or above, and they were all senior professional. The average age and professional experience were 52.6 ± 5.6 years and 33.3 ± 7.5 years, respectively. The average expert authority coefficient was 0.99 ± 0.02, and 100% of respondents completed the questionnaire. Kendall’s W ranged from 0.00 to 0.23, and all final items achieved mean validity ratings exceeding 4.00 on a 5-point Likert scale. These metrics verify sufficient content validity and reliability for subsequent data collection (22).

#### 2.3.2 Final questionnaires

Two structured questionnaires were created to assess IHT practices in different perspectives. The head nurse questionnaire contained three components: (1) The operational characteristics of the unit, including unit type classification, the medical staff’s demographics, and annual volumes of hemodialysis therapy, intrahospital transports, and documented PSIs from January to December 2024. Official nursing documentation provided data on transfers and safety incidents. (2) Demographic information on the head nurse. (3) Clinical practice procedures related to the IHT of hemodialysis patients, such as the formation of institutional transfer regulations, the availability of transfer-specific training, the application of standardized risk assessment with specified criteria, the composition of transport teams, and the execution of quality control activity. There were two sections to the clinical nurse questionnaire: (1) Participating nurses’ demographic data and (2) Self-reported experiences with various PSI types documented in nursing records. The ICPS framework, released in 2009, was used for incident classification (4). Within this framework, individual incidents episodes can be categorized into multiple incident types to capture their multifaceted nature. For instance, both medication-related and equipment-related incident categories would apply to an incident involving improper infusion pump configuration that resulted in anticoagulant overdose and coagulation disorder.

### 2.4 Data collection and procedure

Digital questionnaires were distributed through the Chinese Nursing Association’s Blood Purification Specialty Committee and its affiliated provincial and municipal chapters. Survey distributors transmitted access QR codes to designated healthcare institutions under their control, enabling direct electronic access to the digital platform. To encourage informed participant engagement, each questionnaire included an introduction detailing the study’s goals, significance, completion procedures, and key components. Participants accessed and completed the questionnaire via either desktop computers or mobile devices. All submissions were anonymized and securely stored on the server.

Multiple precautions were implemented to ensure data integrity throughout the collection process. (1) Technical restrictions prevented repeated data entry by limiting each IP address to a single legitimate submission. (2) The online platform disabled the submit function until all required fields contained valid responses. (3) Automated prompts informed respondents of any incomplete or inconsistent entries during completion, allowing real-time correction. (4) The research team continuously tracked submission rates and response quality through a secure backend analytics system.

After data collection, two independent researchers conducted a thoroughly review and cleaning of the dataset. Respondents were contacted directly by phone or email to clarify any unclear or dubious entries. A senior researcher’ third-party adjudication was necessary to settle persistent disagreements between the two primary reviewers. Survey responses were classified as valid only when they met all the following criteria simultaneously, including absence of recording errors, complete response to all items, and internal logical coherence across related questions. Questionnaires that failed to satisfy these validity requirements were excluded from subsequent analyses.

### 2.5 Data analysis

All statistical analyses were performed using IBM SPSS Statistics, version 26.0 (IBM Corp., Armonk, NY, United States). Descriptive statistics were employed to summarize the sample characteristics, with categorical variables presented as frequencies (n) and percentages (%). Continuous variables are reported as mean (standard deviation) or median (interquartile range, IQR) depending on normality testing results. The outcome variable was dichotomized as a binary indicator of safety event occurrence during IHT of hemodialysis patients. Univariate associations between candidate variables and the outcome were assessed using chi-square (χ^2^) tests, with a threshold of *p* < 0.05 determining inclusion in multivariate analysis. A binary logistic regression model employing backward stepwise elimination identified the relevant factors of safety incidents, and all variables achieving statistical significance were entered into the model. Model fit was assessed using the Hosmer-Lemeshow goodness-of-fit test. Effects are expressed as odds ratios (OR) with corresponding 95% confidence intervals (CI). Statistical significance was defined as a two-tailed *p*-value less than 0.05 throughout all analyses. All analyses adhered to standard epidemiological reporting guidelines for observational studies.

Two derived variables were created to standardize staffing density within each unit. Physician density = (total full-time physicians/total hemodialysis patients transferred within the hospital) × 10. Nurse density was calculated similarly using full-time nursing staff counts. Both calculations used data reported during the calendar year 2024 as the reference period for patient transfers.

### 2.6 Ethical considerations

This study was performed in line with the principles of the Declaration of Helsinki. Approval was granted by the Ethics Committee of Peking University First Hospital (Approval No. 2025R0057-0003). Every qualified clinical nurse and head nurse who was eligible to participate in the study gave their electronic informed consent before completing the questionnaire. Every participant was told that no private information was gathered, and the study process adhered to strict guidelines to guarantee anonymity, privacy, and confidentiality.

## 3 Results

### 3.1 Participant characteristics

This study involved 363 head nurses and 3,232 clinical nurses from tertiary public hospitals in 31 official administrative regions of the People’s Republic of China (Table 1). Unit-level service capacity was defined by the total number of hemodialysis operations and intrahospital transports per unit in 2024. The median hemodialysis caseload was 16,587.00 (IQR: 1,560.00–37,839.00, range: 2–600,000), and the median intrahospital transports were 504.00 (IQR: 50.00–1,600.00, range: 1–32,500).

**Table 1:** Baseline characteristics of head nurses and clinical nurses [*n* (%)].

Characteristics		Head nurses (*n* = 363)	Clinical nurses (*n* = 3,232)
Gender	Male	20 (5.51%)	302 (9.34%)
	Female	343 (94.49%)	2,390 (73.95%)
Age	≤30 years	34 (9.37%)	961 (29/73%)
	30 ~ 40 years	124 (34.16%)	1706 (52.78%)
	>40 years	205 (56.47%)	565 (17.48%)
Educational background	Junior college and below	30 (8.26%)	359 (11.11%)
	Undergraduate degree	310 (85.40%)	2,821 (87.28%)
	Master’s degree or above	23 (6.34%)	52 (1.61%)
Professional title	Junior	36 (9.92%)	1,329 (41.12%)
	Intermediate	152 (41.87%)	1746 (54.02%)
	Senior	175 (48.21%)	157 (4.86%)

ICU, intensive care unit.

### 3.2 Clinical practice of intrahospital transport

The proportions of doctors, nurses, medical assistants, and family members involved in intrahospital patient transports were 72.18, 91.74, 56.47, and 80.99%, respectively. The units’ clinical practice actions included pre-transfer risk evaluations (87.88%), development of transit regulations (73.83%), professional training (61.71%), and medical quality control (53.72%). Furthermore, creating multidisciplinary teams (73.85%), setting safe standards (85.64%), holding regular quality improvement meetings (78.97%), offering on-site supervision during transport (67.18%), and assigning transport liaison positions (46.15%) were critical quality control measures.

### 3.3 Nature and type of safety incidents

A total of 209 participating units reported 268 safety incidents out of 135,459 IHT cases involving hemodialysis patients, and 28.23% of these units experienced at least one incident throughout the year. Hemodialysis facilities reported 81.72% of all incidents, general wards (including the nephrology, urology, and infectious diseases wards) reported 17.91%, and the emergency department recorded 0.37%. Based on the investigation of 3,232 clinical nurses, medical device/equipment malfunction and patient accidents were the main sources of incidents reported and are shown in Table 2.

**Table 2:** Main types and its reported cases of patient safety incidents (*n* = 3,232).

Event types of ICPS	Main patient safety incidents	Number	Composition ratio (%)
Clinical administration	Incorrect patient identification Delay/cancelation of transport Loss/omission of specimens, personal belongings, etc.	1,352	41.83%
Clinical process/procedure	Pressure injuries Unplanned extubation Dressing not securely adhered	1,182	36.57%
Medication/IV fluids	Intravenous infusion extravasation/infiltration Improper flow rate Medication administration error	1,346	41.65%
Blood/blood products	Bleeding/hematoma at vascular access site	1,420	43.94%
Nutrition	Hypoglycemia	1	0.03%
Oxygen/gas/vapor	The oxygen tube/ventilator tubing is not properly connected to the patient or the gas source Oxygen source exhaustion	1,370	42.39%
Medical device/equipment	Blockage, dislodgement, or disconnection of tubing (e.g., vascular access, urinary catheters, etc.) Power insufficiency Equipment malfunction	1,976	61.14%
Behavior	The portable oxygen therapy device is not turned on The monitor probes are not properly connected to the patient	1,165	36.05%
Patient accidents	The patient fell Fell out of bed	1,462	45.24%
Infrastructure/building/fixtures	Malfunctions in elevators, wheelchairs, and other equipment	2	0.06%
Resources/organizational management	Failure to prepare adequate equipment (such as monitors, patient stretcher, etc.)	1,337	41.37%

ICPS, the international classification for patient safety.

### 3.4 Relevant factors of safety incidents

As detailed in Table 3, several factors were associated with safety event occurrence. These included the head nurse’s educational background, the involvement of medical assistants in the transport process, the monthly hemodialysis caseload and transfer volume, the density of physicians and nurses, and the presence of formal transfer regulations and medical quality control actions.

**Table 3:** Univariate analysis of the variables that influence safety incidents (*n* = 363).

Variables		Not occurred (*n* = 305)	Occurred (*n* = 58)	χ^2^	*P*
Head nurse professional title	Junior	26 (8.52%)	10 (17.24%)	5.149	0.076
	Intermediate	133 (43.61%)	19 (32.76%)		
	Senior	146 (47.87%)	29 (50.00%)		
Head nurse educational background	Junior college or below	19 (6.23%)	11 (18.97%)	13.065	0.001*
	Undergraduate degree	269 (88.20%)	41 (17.38%)		
	Master’s degree or above	17 (5.57%)	6 (10.34%)		
Management experience of head nurse	≥10 years	161 (52.79%)	27 (46.55%)	0.759	0.384
	<10 years	144 (47.21%)	31 (53.45%)		
Involvement of physician	No	86 (28.20%)	15 (25.86%)	0.132	0.716
	Yes	219 (71.80%)	43 (74.14%)		
Involvement of nurse	No	27 (8.85%)	3 (5.17%)	0.444^#^	0.260
	Yes	278 (91.15%)	55 (94.84%)		
Involvement of medical assistant	No	141 (46.23%)	17 (29.31%)	5.675	0.017*
	Yes	164 (53.77%)	41 (70.69%)		
Involvement of family member	No	59 (19.34%)	10 (17.24%)	0.140	0.708
	Yes	246 (80.66%)	48 (82.76%)		
Monthly hemodialysis caseload	<1,000	141 (46.23%)	16 (27.59%)	16.337	<0.001*
	1,000 ~ 3,000	98 (32.13%)	15 (25.86%)		
	>3,000	66 (21.64%)	27 (46.55%)		
Monthly transfer volume	<100	208 (68.20%)	22 (37.93%)	19.500	<0.001*
	100 ~ 300	71 (23.28%)	25 (43.10%)		
	>300	26 (8.52%)	11 (18.97%)		
Physician staffing density	<1	194 (63.61%)	52 (89.66%)	15.138	<0.001*
	≥1	111 (36.39%)	6 (10.34%)		
Nurse staffing density	<1	153 (50.16%)	45 (77.59%)	14.781	<0.001*
	≥1	152 (49.84%)	13 (22.41%)		
Presence of regulations	No	86 (28.20%)	9 (15.52%)	4.055	0.044*
	Yes	219 (71.80%)	49 (84.48%)		
Transit risk assessment	No	40 (13.11%)	4 (6.90%)	1.769	0.184
	Yes	265 (86.89%)	54 (93.10%)		
Professional training	No	101 (33.11%)	14 (24.14%)	1.814	0.178
	Yes	204 (66.89%)	44 (75.86%)		
Presence of medical quality control actions	No	155 (50.82%)	13 (22.41%)	15.816	<0.001*
	Yes	150 (49.18%)	45 (77.59%)		

#Fisher’s Exact Test as 1 cell have expected count less than 5. *P < 0.05.

Binary logistic regression analysis was performed on variables that demonstrated statistical significance in univariate analyses to identify relevant factors of PSIs. All candidate variables were coded as follows: head nurse educational background (college degree or below = 1; bachelor’s degree = 2; master’s degree or above = 3), medical assistant involvement during transport (no = 0; yes = 1), monthly hemodialysis caseload (<1,000 = 1; 1,000–3,000 = 2; >3,000 = 3), monthly transfer volume (<100 = 1; 100–300 = 2; >300 = 3), physician staffing density (<1 = 0; ≥1 = 1), nurse staffing density (<1 = 0; ≥1 = 1), presence of transfer regulations (no = 0; yes = 1), and medical quality control actions (no = 0; yes = 1).

The independent variables significantly predicted the occurrence of PSI, as indicated by the chi-square statistic of χ^2^ = 348.34 (df = 8, *p* < 0.001) obtained from the omnibus testing of model coefficients. The model’s explanatory power was moderate, with Nagelkerke *R*^2^ = 0.232 and Cox and Snell *R*^2^ = 0.136. Adequate model calibration was shown by the Hosmer-Lemeshow goodness-of-fit test (χ^2^ = 11.256, df = 8, *p* = 0.188). Table 4 demonstrates that a monthly hemodialysis caseload exceeding 3,000 procedures (adjusted OR = 2.580, 95% CI: 1.198–5.556) and the presence of medical quality control actions (adjusted OR = 3.540, 95%CI: 1.604–7.814) were associated with significantly elevated risk of safety incidents.

**Table 4:** Logistic regression analysis of factors associated with safety incidents (*n* = 363).

Variables	β	Wald	*P*	OR	95% CI
Head nurses educational background					
College degree or below				1.00	
Bachelor’s degree	−0.487	0.989	0.320	0.615	0.236–1.604
Master’s degree or above	0.649	0.833	0.362	1.913	0.475–7.706
Involvement of medical assistant					
No				1.00	
Yes	0.336	0.971	0.324	1.400	0.717–2.733
Monthly hemodialysis caseload					
<1,000 procedures				1.00	
1,000 ~ 3,000 procedures	0.439	1.060	0.303	1.551	0.673–3.578
>3,000 procedures	0.948	5.867	0.015	2.580	1.198–5.556
Monthly transfer volume					
<100 procedures				1.00	
100 ~ 300 procedures	0.417	0.800	0.371	1.518	0.608–3.788
>300 procedures	0.487	0.779	0.377	1.628	0.552–4.806
Physician staffing density					
<1				1.00	
≥1	−0.928	2.320	0.128	0.395	0.120–1.305
Nurses staffing density					
<1				1.00	
≥1	−0.283	0.252	0.616	0.754	0.249–2.276
Presence of regulations					
No				1.00	
Yes	0.041	0.008	0.929	1.042	0.421–2.580
Presence of medical quality control actions					
No				1.00	
Yes	1.264	9.791	0.002	3.540	1.604–7.814
Constant term	−2.731	11.683	<0.001		

## 4 Discussion

### 4.1 Findings on transport-related safety incidents

Among 135,459 IHT experiences involving hemodialysis patients in 2024, this study found 268 safety incidents, the majority of which included patient accidents and medical devices or equipment malfunction. Hemodialysis facilities reported 81.72% of these events because they serve as major receiving units for dialysis inpatients. The fact that hemodialysis centers are typically physically separate from transmitting units (e.g., general wards, intensive care units, and emergency departments) in China is reflected in this distribution, which may introduce unpredictability in anticipated transit times. Extended transit times have been linked to increased likelihood of unfavorable outcomes, conversely, streamlining transportation processes reduced the mean transit time from 39.0 to 27.0 min, which in turn correspondingly decreased incidence of adverse events from 13.3 to 3.3% (*p* < 0.05) (23). In order to reduce transit time, hospital officials ought to consider putting in place priority transport lanes or special transport elevators. Additionally, optimal transportation routes should be identified through systematic evaluation and validation procedures. To enhance overall patient safety outcomes, admission and discharge convenience should be integrated into proactive spatial layout planning when designing new hemodialysis facilities.

Barriers pertaining to reporting culture were also identified. The risk of safety incidents was 3.54 times higher for units implementing quality control actions for IHT (95% CI; 1.60–7.81; *p* = 0.002). This was interpreted as consistent with higher reporting rates under active reporting initiatives, thereby providing estimates closer to actual incidence. Patient safety event reporting is often characterized by underreporting and bias toward events causing harm and against near-miss or no-harm events for various reasons, such as immature patient safety surveillance systems (24), inconsistent reporting regulations, or an unwelcoming reporting environment (25). Since most incidents occur during transitional processes, certain hidden events cannot be automatically recorded when medical staff are either not present at the incident scene or unable to recognize near misses. Selective reporting, where many institutions and healthcare providers document both preventable and non-preventable adverse events with significant underreporting of non-harm events, is common in various locations and cultures (26). Furthermore, predominantly utilizing accountability systems may foster an atmosphere of fear among medical staff, making them reluctant to voluntarily report errors due to concerns about resulting repercussions (27, 28). As a result, voluntary reporting systems usually report mainly negative occurrences, with low safety event reporting rates and reporting cultures that are more likely to identify incidents with unfavorable consequences (29). In order to prevent individual subjective reporting intentions and behaviors from interfering with compliance, medical institutions should link patient transport safety with fair performance appraisal systems and optimize patient safety surveillance systems by fully integrating electronic health records with the transition process (30).

### 4.2 Standardization of policies and quality control actions

Standardized policies provide healthcare personnel with clear operational procedures and defined duties. According to our findings, 73.83% of units have implemented transport regulations for hemodialysis patients, avoiding ambiguous staff responsibilities, disorganized transport operations, incomplete information handover, and increased patient safety hazards (9). Hospital administrators ought to consult relevant standards to refine the process for intrahospital patient transfers. The following actions could greatly improve transport quality, especially in preventing unfavorable events caused by human error, including simulating IHT scenarios (31), implementing transport verification checklists (15), performing a proactive risk assessment (32), and enacting structured handover protocols (33).

It is noteworthy that transport-related safety incidents were more inclined to happen at times of high transfer volume (34) as the family members can serve as an important source to offer additional assistance. There are differences in policies among various countries and regions regarding whether family members should be allowed to accompany transitions (1) while their participation reached 80.99% in our study. WHO’s Global Patient Safety Report 2024 (35) advocated that patients and their families are key partners in creating and executing policies and action plans for patient safety, in line with the theme of the 2023 World Patient Safety Day. In many transfer scenarios, patients may not be able to make decisions for themselves due to disease or cognitive impairment, and family members or legal representatives may be required to engage in patient handovers, particularly in emergency settings. However, there are also some concerning circumstances, such as the family’s intense worry and anxiety for the patient’s well-being, making it difficult to effectively carry out routine medical treatments (2). Therefore, if possible, allow family members to accompany the patient during IHT depending on how effectively medical matters are communicated with them before transportation based on the standardized policy.

Furthermore, to promote the standardization and normalization of transport procedures, relevant quality control based on transport protocol is an effective measure to reduce PSIs. However, only 53.72% of units in this study performed quality improvement during IHT, demonstrating that quality control remained a weak link in current patient safety management systems. Among quality control measures, the percentage to define the safe standards (85.64%) was highest, which showed that most units continued to prioritize the formation of quality evaluation criteria.

### 4.3 Workload intensity and resource distribution

The quality of healthcare services is closely linked to unequal medical resource distribution. Our findings demonstrated that the risk of safety incidents occurring increased by 2.58 times (95% CI, 1.20–5.56, *p* = 0.015) when the monthly hemodialysis caseload exceeded 3,000 procedures, suggesting that excessive workload may compromise healthcare quality. Regarding human resource allocation, related guidelines (2) recommended deploying the number of healthcare professionals and support staff with qualifications commensurate with the patient’s severity of illness. In our study, physicians and nurses participated in transport at rates of 72.18 and 91.74%, respectively. Alizadeh Sharafi et al. (36) suggested that the charge nurse should serve as a critical member in the transfer team, primarily responsible for information handover while acting as a coordinator and organizer to ensure patient safety during transportation. When transporting patients with tracheal intubation, those with hemodynamic instability, or those anticipated to require emergency intervention, at least one physician with appropriate qualifications and competency must accompany the transport, considering that they may compensate for shortcomings in incident response and emergency abilities, thereby lowering safety incidents (2).

Statistics indicated that the average transit time per critically ill patient ranged from 13.50 to 39.00 min (37), and a total transport duration of more than 60 min was a high-risk factor for adverse transport events (OR = 7.62, 95% CI: 3.38–17.19) (38). As charge nurses undertake the primary responsibility of patient transportation, it means they must expend additional time and energy to juggle transport duties when transport tasks become excessive. They were prone to developing physical fatigue, anxiety, depression, and even fear and dread, which were compounded by potential factors such as human resource shortages, lack of relevant knowledge, and environmental distractions. This compromised their attention to patient conditions, the precision of their procedures, and their responsiveness to emergencies, thereby undermining the safety of in-hospital patient transfers.

Hemodialysis therapy is a complex and highly specialized clinical process that necessitates accurate assessments of the patient’s condition, dialysis protocols, and complications connected to dialysis both before and after treatment. Clinical errors and adverse outcomes may result from healthcare professionals’ inadequate understanding and core competencies of patient safety. Yet comprehensive integration of patient safety into health professional education and training remains limited globally (35), and systematic training was a critical measure to enhance healthcare personnel’s transport competence and safety culture (34). But our finding showed that only 61.71% of units conducted professional training, which was far from meeting clinical demands. Thus, hospitals should implement targeted strategies that can bridge the medical staff’s competence gap to enhance the transition safety.

### 4.4 Strengths and limitations

There were several strengths to this study. In order to ensure the validity and reliability of the questionnaire, we conducted a review of pertinent literature, clinical observations, and expert consultations, which ensured the rigor of the study. Second, to make a good generality, we employed a multistage convenience sampling strategy and surveyed 4,253 registered nurses from 31 official administrative regions across China. Third, this is the first study focused on the precise profile of safety incidents occurring during IHT among hemodialysis patients, particularly within China’s healthcare delivery model, where hemodialysis services are mainly provided through hospital-based centers rather than independent facilities. Finally, in addressing the status of clinical practices from a nurse’s perspective, this study complements existing sources of safety intelligence with the potential for service improvement.

The limitations of this study were as follows. First, its cross-sectional design precludes causal inference, and finding require validation through prospective longitudinal follow-up. While the ICPS serves as a guiding framework for prevention and organizational learning, the absence of standardized incident classification data from patient transport records across participating institutions constrained our ability to conduct detailed categorical analyses of safety incidents. Second, regarding model selection, the use of simple logistic regression may have underestimated standard errors given the hierarchical structure of the data, potentially affecting the precision of effect estimates. Finally, although the structured questionnaires were initially verified to have sufficient content validity and reliability through the Delphi approach for subsequent data collection, further optimization is still needed with a larger sample size and surveys conducted in multiple countries or regions.

## 5 Conclusion

This study comprehensively profiled safety incidents related to IHT among hemodialysis patients in Chinese tertiary hospital, providing essential evidence for the formulation of targeted safety standards. These findings reveal that policy standardization and targeted resource allocation are vital to improving safety outcomes in hospital-based hemodialysis care. To improve the reporting rate of safety events, evidence-based strategic interventions are urgently required, including full integrating electronic health records with transition processes, fostering non-punitive safety reporting cultures and embedding patient safety into health professional education. These multi-dimensional interventions, which cover the operational, technical and cultural aspects of patient safety in complex clinical environments, should be prioritized in future clinical practice. Where possible, local researchers could attempt to validate these findings in their countries and institutions where hemodialysis services are predominantly delivered through hospital-based centers rather than independent facilities, taking into account factors such as hemodialysis caseload, the relevant policies, reporting culture and so on.

Nevertheless, this study only focused on practitioner and operational factors in the Chinese hemodialysis context and failed to fully explore core confounding variables that directly affect IHT safety. These unaddressed factors include the severity of the patient’s condition, unique hemodialysis clinical characteristics, and key transit-related indicators such as transport duration, transport distance, and the time of transport. Future study should adopt prospective designs and in-depth research how to integrate Electronic Health Records to capture these real-time operational variables to build a better risk stratification tool. Moreover, standardized protocols for IHT should be developed, and their clinical efficacy evaluated once contributing factors are fully identified.

## Data Availability

The datasets presented in this article are not readily available because the authors confirm that the data supporting the findings of this study are available within the article. The comprehensive data that support the findings of this study are available from the corresponding author, upon reasonable request. Requests to access the datasets should be directed to Yuqing Chen, cyq@bjmu.edu.cn.
